# Unveiling a Near‐Total Rear Passivation for High Performance of Ultra‐Thin ACIGS

**DOI:** 10.1002/advs.77285

**Published:** 2026-09-27

**Authors:** Xavier L. Pinheiro, António J. N. Oliveira, André F. Violas, Kevin Oliveira, Paulo A. Fernandes, Marika Edoff, Pedro M. P. Salomé, Jennifer P. Teixeira

**Affiliations:** ^1^ INL – International Iberian Nanotechnology Laboratory Avenida Mestre José Veiga Braga Portugal; ^2^ Departamento De Física da Universidade de Aveiro Campus Universitário de Santiago Aveiro Portugal; ^3^ CICECO Aveiro Institute of Materials University of Aveiro Campus Universitário de Santiago Aveiro Portugal; ^4^ CIETI ISEP Instituto Politécnico do Porto Portugal; ^5^ Department of Materials Science and Engineering Division of Solar Cell Technology Ångström Laboratory Uppsala University Uppsala Sweden

**Keywords:** ACIGS, full‐layer passivation, silicon oxide (SiO_x_), ultra‐thin

## Abstract

Thin‐film Cu(In,Ga)Se_2_ solar cells are a key photovoltaic technology for addressing global energy and environmental challenges. Reducing absorber thickness lowers manufacturing cost and critical raw material consumption but introduces optical and electrical losses due to incomplete light absorption and increased rear interface recombination. Rear recombination can be mitigated through Ga grading and/or rear contact passivation, for example using tunnel oxide as in c‐Si TOPCon technology. In this work, a full coverage plasma enhanced chemically vapor deposited 8 nm porous SiO_x_ passivation layer was implemented in ultra‐thin (Ag,Cu)(In,Ga)Se_2_ (ACIGS) solar cells and compared with patterned passivation layers (∼70% coverage) and an unpassivated reference. Standard current‐voltage and external quantum efficiency characterization methods show that the complete passivation yielded the highest performance, achieving 16.4% efficiency, a 2% absolute improvement over the reference. Patterned samples showed enhanced performance to a lower extent. Elemental and structural analysis showed that the SiO_x_ layers remained intact after 550°C deposition of the 700 nm ACIGS absorber. Notably, a MoSe_2_ interfacial layer formed between the full SiO_x_ layer and Mo contact, likely due to Na and/or Se diffusion through SiO_x_ pores. Combining near‐complete passivation and the ohmic MoSe_2_ contact may explain the improved performance.

## Graphical abstract

Ultra‐thin solar cells of (Ag,Cu)(In,Ga)Se_2_ (ACIGS) suffer from rear interface recombination, limiting their performance. To tackle it, a full coverage plasma enhanced chemically vapor‐deposited 8 nm porous SiO_x_ passivation layer was implemented and evaluated. It is shown that a combination of the near‐complete passivation from silica and the formation of MoSe_2_ below may explain the higher performance.

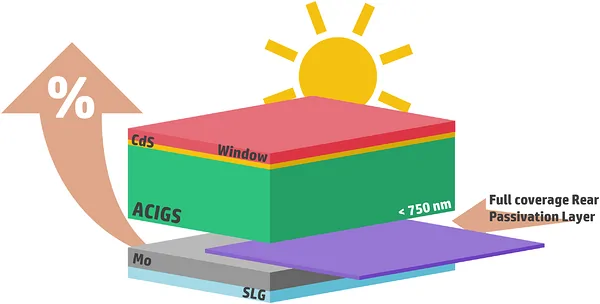

## 1 Introduction

The relentless pace of technological advancement, the increasing global population, climate change, and the world's geopolitical context have fuelled a surge in energy demand in the past few years. Global energy demand increased by 2.3% in 2024, while electricity demand grew even more rapidly, by 4.3%, approximately twice the rate of total energy demand [1]. This impressive and challenging requirement to fulfil can be partially answered with one of the primary sources of clean energy—photovoltaics (PV), allowing it to also meet sustainable energy targets defined by the European Union [2]. Specifically, Building Integrated PV (BIPV) brings energy production directly to the consumer and having the advantage of decreased land usage [3]. In late 2024, the BIPV market accounted for 2 GW of installed power globally, while standard PV was above 1.8 TW [4, 5, 6]. In order to increase the BIPV footprint, PV technologies must meet the necessary criteria of efficiency, stability, weight, flexibility, and aesthetics [7]. Despite wafer‐based crystalline silicon (Si) dominance, specifically monocrystalline, over the PV market – 97% at the end of 2025 [8], thin‐film technologies can be key players in BIPV applications. Specifically, Cu(In,Ga)Se_2_ (CIGS) solar cells provide several advantages, such as great thermodynamic stability [9], high absorption coefficient [10], and mature commercialization in flexible substrates [11]. Moreover, it holds a world record efficiency of 23.6% [12, 13] and has a low energy payback time [14]. One extra step can be taken if, instead of a thin‐film (∼2000 nm thick absorber), the ultra‐thin‐film range in CIGS is considered (≤ 750 nm). Such alteration allows to decrease the production costs and time, increase throughput, and reduce the usage of critical raw materials, meeting sustainable goals [2, 15]. The ultra‐thin regime further enables the production of lightweight and flexible modules, easing their application in BIPV, which will reduce the building´s CO_2_ emissions, leading to a net‐zero scenario. However, such drastic reduction in the absorber thickness has led to a performance loss from 23.6% to 16.2% or 18.2% for 750 nm, considering the full or active area, respectively, and to 15.2% for 490 nm [16, 17]. The main causes of such loss are incomplete light absorption, with a severe decrease for thickness values below 500 nm, and rear interface recombination, which becomes more impactful at this level [18, 19, 20]. Two main strategies have been employed to mitigate recombination at the rear interface: (i) Ga grading with a higher content at the rear, which is a demanding task in an ultra‐thin absorber due to the challenging elemental diffusion control and reproducibility [16, 21], and (ii) the addition of a nanostructured passivation layer [22]. The dielectrics used as passivation layers, alumina (AlO_x_), silica (SiO_x_), titania (TiO_x_), and hafnia (HfO_x_) [23, 24, 25] allow to increase the performance of the overall solar cell by reducing the impact of rear interface defects, but these have to be patterned, e.g., with lines or holes, to allow a direct contact between the absorber and the metallic back contact [19, 26, 27, 28, 29, 30] since they commonly are insulators. Nevertheless, it may be possible to implement a full‐layer strategy by replicating what has been done in Si technology [31, 32, 33]. The implementation of carrier‐selective contacts at critical interfaces provides passivation, conductivity, and selectivity, reducing recombination losses and enhancing the charge carrier collection at the corresponding interfaces [34, 35]. It is no easy feat to provide all these requirements with one single layer, so architectures combining passivation (dielectrics), selectivity (e.g., transition metal oxides), and conductivity (e.g., transparent conductive oxides) layers have been tested in Si solar cells [31, 32]. Regarding CIGS solar cells, some progress has been made by applying selective layers at the front and rear interfaces with proven performance increase [36, 37, 38, 39, 40]. While the development and implementation of selective contacts may require an individual thin layer, or even the combination of several distinct thin layers, the integration of one single ultra‐thin layer may be capable of providing similar benefits to performance. Tunnel oxide passivated contact (TOPCon) is one of the most promising crystalline Si solar cell architectures; in this structure, an ultra‐thin (1–2 nm) silicon oxide layer is added to the rear contact, enabling interface passivation and charge transport through tunnelling [41, 42, 43, 44, 45]. TOPCon technology is at a critical point with a proven record efficiency of 27.8% [46], comparable with 27.8% for heterojunction interdigitated back contact (HIBC) solar cells [47] and 24.5% for Passivated Emitter Rear Contact (PERC) [47]. However, creating a passivation layer with such reduced thickness requires highly controllable and conformal deposition techniques, such as Atomic Layer Deposition (ALD) [48, 49]. Furthermore, in CIGS based solar cells an ultra‐thin layer may not be able to provide the necessary passivation effect, depending on the material: for Al_2_O_3_, full passivation layers with 2 and 5 nm still led to a blocking behavior [50, 51, 52]; considering HfO_2_, thickness values below 2.5 nm do not show a blocking behavior but the solar cell performance is not improved [24]. Jian et al. stated that in ultra‐thin passivation layers two transport mechanisms may occur: (i) pure tunnelling and (ii) type ohmic through the pinholes or pores in the oxide layer [41]. Therefore, a porous passivation layer can be implemented in a full‐area architecture, possibly delivering passivation effects while maintaining charge conduction.

The present study tests the possibility of reaching a significant passivation effect in an ultra‐thin (Ag,Cu)(In,Ga)Se_2_ (ACIGS) solar cell with a full coverage thin layer of a porous dielectric. Additionally, it is compared with a conventional device with no passivation scheme, as a reference, and two line‐patterned passivated devices around 70% coverage and different thickness values. Silica (SiO_x_), deposited by plasma‐enhanced chemical vapor deposition (PECVD), was chosen as a passivating material due to its effective rear interface passivation, and the thickness was around 8 nm to maximize the passivation effect, as previously reported [19, 24]. Current density‐voltage (JV) and external quantum efficiency (EQE) measurements were performed under standard conditions for solar cell characterization. The results show a clear light‐to‐power conversion efficiency improvement through the application of any of the studied schemes, with the highest efficiency for the porous full‐layer. Through electron microscopy, a uniform distribution of MoSe_2_ was identified at the SiO_x_/Mo interface of the 8 nm patterned and porous full layers, which was assumed to be formed during the diffusion of Se and Na through the pores of the dielectric layer. The higher performance is attributed to the combination of near 100% passivation area with the ohmic‐type contact of the Mo/MoSe_2_/(SiO_x_)/ACIGS enabled by the pores.

## 2 Experimental Section

**FIGURE 1:**
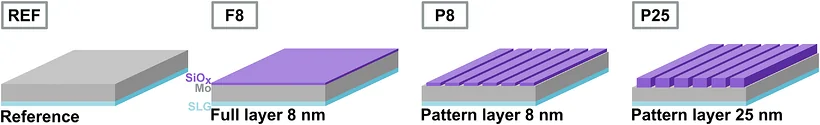
Graphical representation of the prepared substrates with, respectively, no passivation layer (REF), a full 8 nm SiO_x_ layer (F8), and a line pattern SiO_x_ layer of 8 (P8) and 25 nm (P25).

The solar cells fabrication started with 1.1 mm thick Soda‐Lime Glass (SLG) substrates with a sputtered 350 nm Mo (molybdenum) layer on top. Four devices were fabricated, with the substrates depicted in Figure 1: a conventional reference (REF) with no passivation layer; and three passivated samples in which two have a porous 8 nm SiO_x_ layer, one full coverage (F8) and the other line patterned (P8), and the latter is a 25 nm line patterned SiO_x_ layer (P25). The SiO_x_ layers were deposited by PECVD using an SPTS MPX CVD system, with a plasma power of 30 W at 13.56 MHz, a substrate temperature of 300°C, using SiH_4_ and N_2_O precursor gases, and 0.9 Torr working pressure. Majee et al. reported silica films with high porosity levels (25%) with similar PECVD deposition parameters [28], so the deposition conditions were the same for all passivation layers, and the thickness was the critical parameter. After this step, the samples were prepared separately, according to the required patterning, while the full‐layer sample was ready for the absorber deposition. The line pattern samples were coated with 600 nm of photoresist (AZ1505) to be exposed using direct write lithography (DWL), a photolithography laser system from Heidelberg – DWL 2000. After exposure, the photoresist was developed in AZ 400K 1:4, revealing the open lines with approximately 900 nm of width and 2800 nm pitch. The exposed lines were then etched by reactive ion etching using an SPTS ICP system with a CF_4_ chemistry. The remaining photoresist was removed from the samples by an acetone bath for 30 min, followed by an isopropanol and deionized water cleaning in an ultrasound bath.

Prior to the absorber deposition, a 15 nm layer of sodium fluoride (NaF) was evaporated on top of the substrates [53]. Subsequently, the ACIGS absorber layers were deposited on all four devices in a single co‐evaporation run at 550°C, resulting in a nominal 700 nm thick layer, compatible with an ultra‐thin solar cell configuration. The composition values of this absorber layer, determined by x‐ray fluorescence (XRF) performed in a Panalytical Epsilon 5, are ([Ag]+[Cu])/([Ga]+[In]) = 0.79, [Ag]/([Ag]+[Cu]) = 0.11, and [Ga]/([Ga]+[In]) = 0.40. An intentional Ga profile was implemented with a high‐Ga and low‐In content near the rear interface. Afterward, the devices were completed with a 50 nm cadmium sulphide (CdS) layer deposited by chemical bath deposition and an optical window of 50 and 200 nm sputtered bi‐layer of zinc oxide (ZnO) i‐ZnO/ZnO:Al, respectively [9]. The individual cells, which have an area of 0.1 cm^2^, were separated via a lithography step and chemical etching of the CdS/i‐ZnO/ZnO:Al stack [54]. No post‐deposition treatments or anti‐reflection coating were applied in the studied devices.

After the passivated substrate preparation, these were characterized by scanning electron microscopy (SEM) through a FEI Nova Nano SEM 650 system with an acceleration voltage of 5 kV. X‐Ray Reflectometry (XRR) analysis was performed on a PANalytical X'Pert PRO Materials Research Diffractometer equipment (Cu Kα x‐ray tube λ = 0.154 nm, 45 kV, and 40 mA) to assess the film density and porosity level. With a complete device, the solar cell elemental variation and Ga grading were measured with a depth profile by Glow‐discharge optical emission spectroscopy (GDOES). Then, the devices were characterized under standard conditions by JV measurements, and EQE was taken at 0 V. Capacitance‐voltage (CV) measurements were also performed with an AC signal with an amplitude of 25 mV and a frequency of 10 kHz. Optical simulations allow us to study the optical impact of the passivated layers on the solar cell performance and, in this way, decouple the possible electrical gain. Hence, the commercial software Ansys|Lumerical was used to run the 3D finite‐difference time‐domain (FDTD) modelling of the solar cell devices in this work [20, 55]. The simulations were designed with the smallest possible mesh size, considering time and memory constraints. Besides, for critical interfaces, a mesh size of 1 nm was considered. The simulation included both refractive index (n) and extinction coefficient (k) values (n(λ) = n+ik), taken from the literature [10, 56, 57, 58]. Cross‐sectional samples from the produced devices were prepared using a FEI Helios NanoLab 450S focused ion beam (FIB) to have electron‐transparent lamellae (thickness < 100 nm) for subsequent scanning transmission electron microscopy (STEM) coupled with energy‐dispersive x‐ray spectroscopy (EDS) analysis. To ensure defect‐free lamella fabrication preserving its condition, a protective layer of platinum was deposited before any etching. Since a gallium (Ga) ion source was used to etch, this element was excluded from the EDS study. High‐angle annular dark‐field (HAADF) STEM images were acquired using a double‐corrected FEI Titan G3 Cubed Themis; images of 2048 × 2048 pixels were recorded using a convergence angle of 21 mrad with pixel dwell time set to 8 µs. EDS‐mapping was performed with the same instrument using a Super‐X EDS detector. Iterative maps of 2048 × 2048 pixels were recorded with a dwell time per pixel of 5 µs at 200 keV, under a current between 150 and 250 pA and collection times of 15 min. The Velox software from Thermofisher Scientific was used for acquiring and processing the STEM‐EDS data. For the acquired elemental composition line scans, the following characteristic x‐ray emission lines were used: Kα for Si and Se, and Lα for Mo. Regarding the x‐ray photoelectron spectroscopy (XPS) analysis, the system ESCALAB 250 Xi Thermo Scientific equipped with a monochromatic Al Kα source (1 487 eV, 500 µm radius spot) was used. The acquired spectra include a low‐resolution Survey to assess all present elements and high‐resolution for quantification of C 1s, Si 2p, O 1s, Ag 3d, Cu 2p, In 3d, Ga 2p, Se 3d, Mo 3p, and Na 1s elemental orbitals.

## 3 Results and Discussion

**FIGURE 2:**
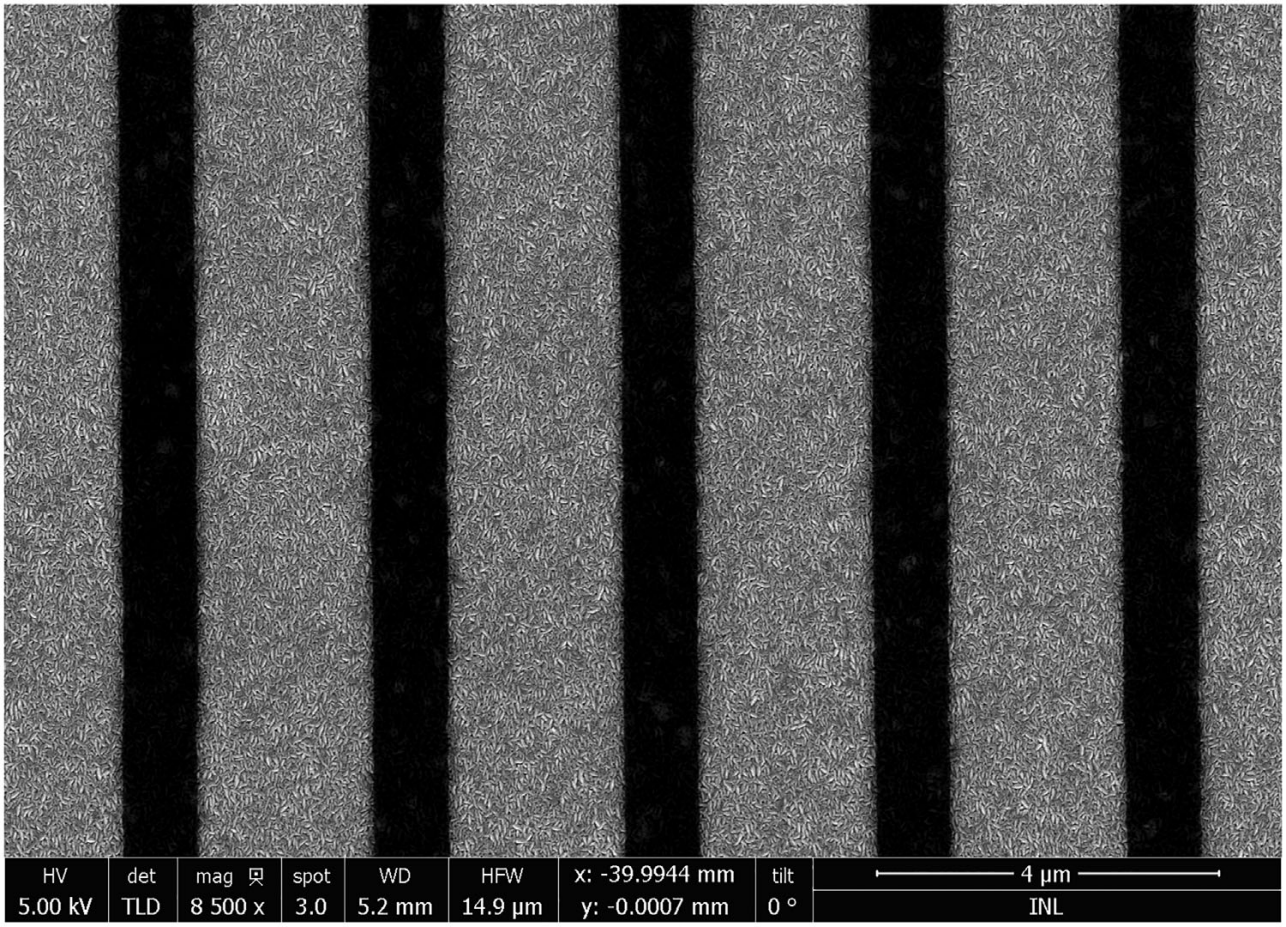
Representative SEM top‐view of the SiO_x_ line pattern of sample P25. The pattern on sample P8 was found identical.

Figure 2 shows a representative SEM top‐view image of the developed line contact passivation scheme, consisting of a direct contact region with the Mo back contact (in black) and dielectric lines for passivation (in light gray). The developed scheme features a passivation line width of 1945 ± 22 nm with a pitch of 2800 nm for both P8 and P25 samples. The contact openings have a width of 855 ± 33 nm, accounting for a passivation area of around 69.5%. Concerning the full porous layer, XRR was used to determine its density and thus porosity. For a similar 20 nm SiO_x_ thin‐film on a Si substrate (chosen to have sufficient interference in the XRR pattern), a density (ρ) of 1.3 g∙cm^−3^ was determined using the critical angle θ_c _ ($\theta_{c\;}\approx \sqrt{2\delta }\propto \sqrt{\rho }$) [59]. The typical density of SiO_2_ deposited through PECVD revolves around 2.3 g/cm^3^ [60], indicating the deposited thin‐film has a porosity level above 40%.

**FIGURE 3:**
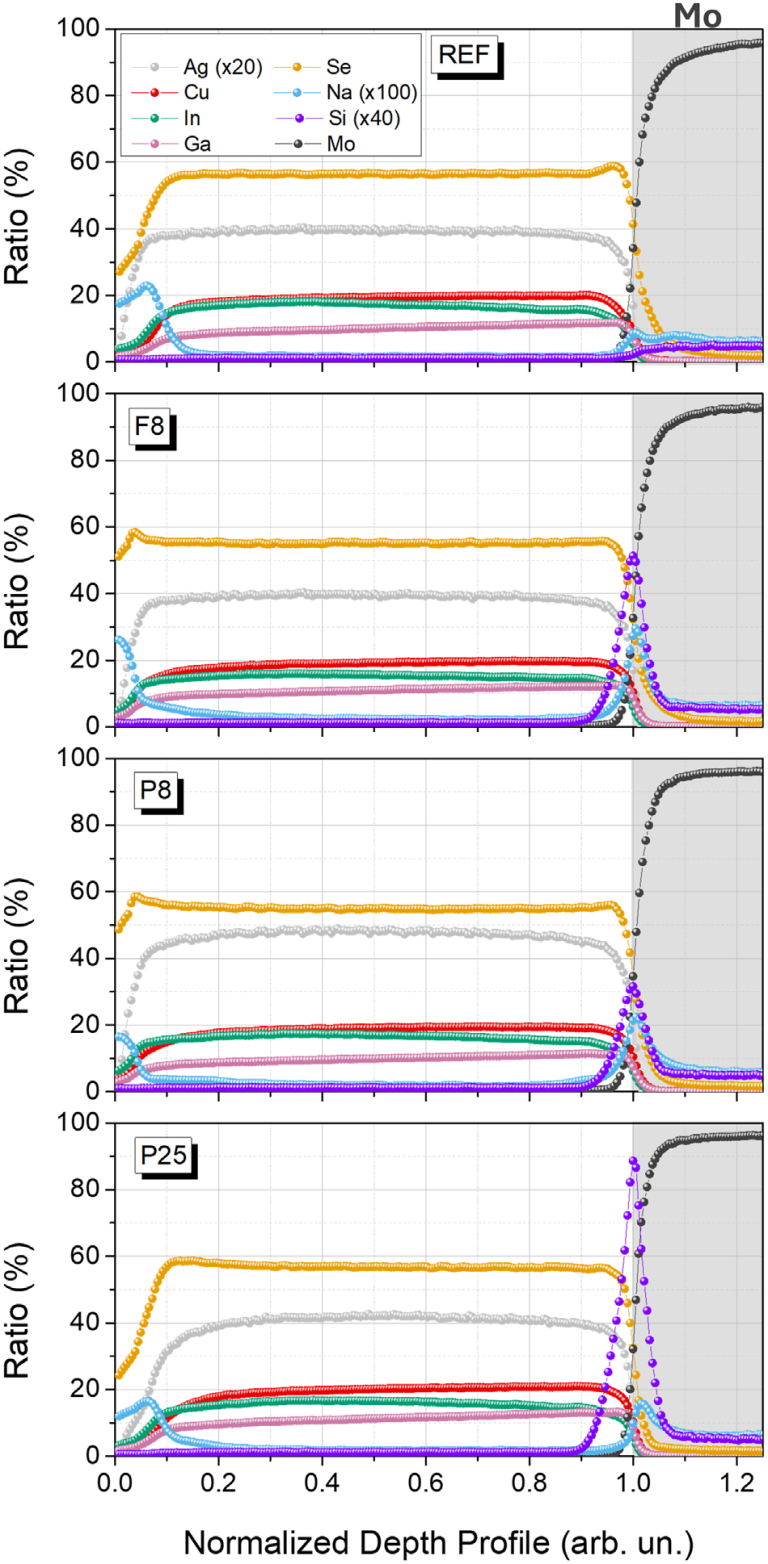
GDOES depth profiling elemental distribution of samples REF, F8, P8, and P25.

The remaining solar cell layers (ACIGS/CdS/ZnO bi‐layer) were deposited on the prepared substrates. The first approach to evaluate the incorporation of the passivation layers, full or patterned, into the ACIGS solar cell architecture was based on an in‐depth elemental analysis. The GDOES elemental plots are shown in Figure 3 for the REF, F8, P8, and P25 solar cells. The topmost layers (CdS/i‐ZnO/ZnO:Al) were chemically etched so the elemental profiling starts at the ACIGS absorber. Regarding the Si signal, associated with the passivation layers, it is possible to observe a Si peak at the interface where the layer is located for all the passivated samples. As expected, the higher the SiO_x_ thickness, the more intense the Si signal. Moreover, despite the same passivation layer thickness value, F8 presents a higher Si signal than P8, which might be related to the higher passivation area attained for the F8 device. Altogether, these results suggest that the passivation layer was confined to the rear interface following the ACIGS deposition, although this analysis does not ensure that the developed schemes remained intact. Another tendency to highlight is related with sodium (Na), where the presence of a Na peak at the rear interface can be attributed to accumulation at the rear interface of the ACIGS for the passivated samples. This result has already been previously reported for passivated ultra‐thin cells with a NaF pre‐deposition layer, since the SiO_x_ layers can partially block its diffusion; however, it is difficult to pin‐point the origin of Na accumulation, either from the NaF or diffusion from the SLG substrate, which is not a crucial point [24]. In all samples, throughout the ACIGS layer thickness, Ag, Cu, and Se are mostly kept constant; for Ga and In, there is a slight increase and decrease, respectively, toward the Mo rear contact. Such linear grading is compatible with the commonly implemented Ga‐profile that increases both open circuit voltage (V_OC_) and fill factor (FF) by repelling photogenerated electrons from the rear interface, further reducing rear recombination [61, 62]. This strategy can be considered one of the first steps to develop a carrier selective contact for ACIGS solar cells since there is an electronic engineering that changes the conductivity of one of the charge carriers and hence the selectivity at the interface. We highlight that this Ga profile is the same in all samples.

**FIGURE 4:**
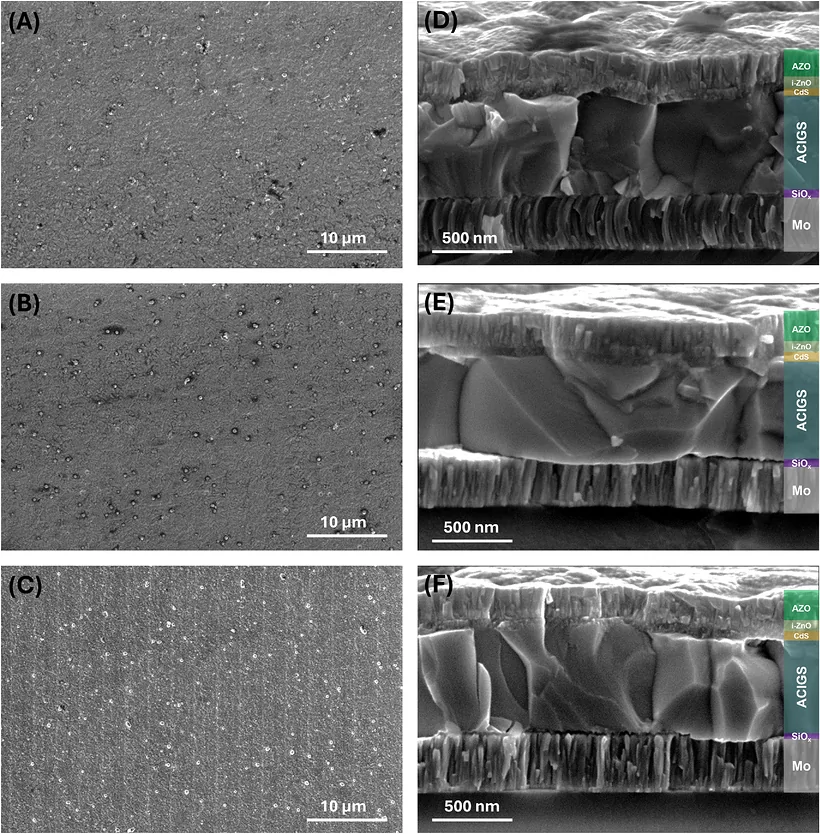
Top‐view SEM images of sample (A) F8, (B) P8, and (C) P25, and SEM cross‐sectional images of sample (D) F8, (E) P8, and (F) P25.

Additionally, the final devices were characterized via SEM top‐view and cross‐section to access the surface and layer's morphology. In Figure 4A to C the top‐view images of the F8, P8, and P25 samples surface are presented, in which a uniform surface morphology is found in Figure 4A arising from the full coverage passivation layer, while for the patterned samples in Figure 4B,C the line pattern can be noted. For sample P25, it is more noticeable since the thickness is higher, resulting in a transference of the pattern to the top layer, but for sample P8, since only 8 nm were used, only a small contrast can be noted. Despite the pattern transfer, in the cross‐sectional characterization of Figure 4E,F, a clean coverage of the buffer and window layers on top of the absorber was attained; therefore, with no significant impact on the optoelectronic properties of the devices. In all samples, the columnar Mo rear contact can be seen, after which the ACIGS absorber is with similar size large grains.

**FIGURE 5:**
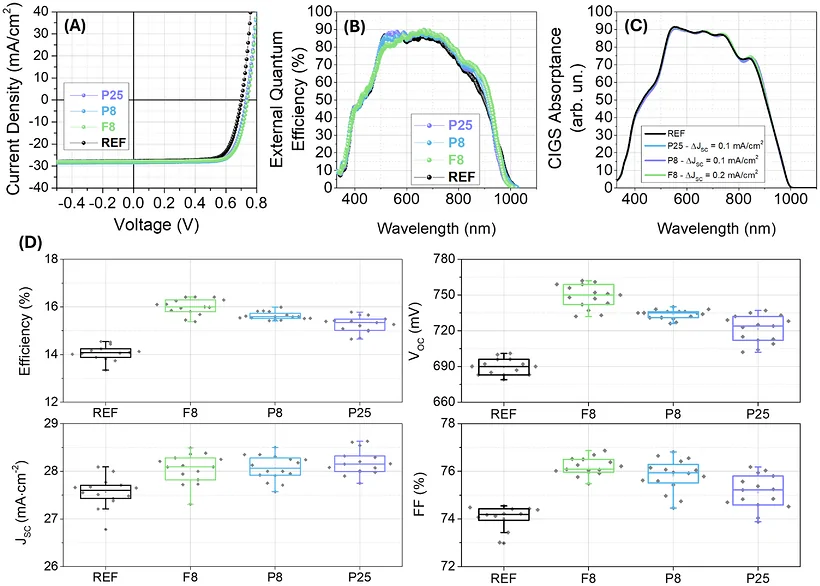
Electrical characterization: (A) current density – voltage (JV) characteristics curve of representative solar cell for all samples, (B) measured (left side) and simulated (right side) external quantum efficiency plots for all four samples, and (D) a statistical representation of the figures of merit obtained from the JV measurements.

The solar cells fabricated with the aforementioned substrates were characterized with JV and EQE, with the representative JV illuminated curves being shown in Figure 5A, and the averaged values from the figures of merit, along with the best cell parameters, presented in Table 1. All devices exhibit diode‐like JV characteristics, with no evidence of significant shunt paths or excessive series resistance. By strict best cell comparison, the highest efficiency value of 16.4% is obtained for the F8 sample, followed by the line pattern samples, P8 and P25, with the REF sample presenting the lowest efficiency value of 14.6%. It is important to note that the value of 16.4% efficiency is in the same range as the current record efficiency for sub‐750 nm cells, which is an indication of an effective coupling of an optimized ACIGS absorber with an efficient rear interface passivation strategy [16, 17]. The same trend is obtained for the average efficiency values. The integration of a rear interface passivation scheme, independently of the architecture, enables for improved V_OC_, FF, and short circuit current density (J_SC_) values, in comparison to the REF device. The highest V_OC_ increase, which corresponded to a value of around 60 mV, was obtained for the F8 sample, which might have arisen from a larger passivation area with respect to the patterned substrates. In order to differentiate the passivation V_OC_ gains, CV characteristics were acquired for each sample, allowing the determination of the net free carrier concentration (N_cv_) at the short circuit condition [63, 64]: 5.1 × 10^15^ cm^−3^ for REF, 6.1 × 10^15^ cm^−3^ for F8, 5.0 × 10^15^ cm^−3^ for P8, and 2.8 × 10^15^ cm^−3^ for P25. With these values, the expected V_OC_ variation from the N_CV_ differences ($\Delta V_{OC-N_{CV}}$) can be calculated for each of the passivated samples [28], and then the effective passivation gain ($\Delta V_{OC-Pass.}=\Delta V_{OC-JV}\;-\;\Delta V_{OC-N_{CV}}$) may be estimated. For the patterned passivation samples, P8 and P25, a Δ*V*_*OC* − *Pass*._ gain of around 45 mV was attained, while for F8 a gain of 55 mV was obtained. A similar effective passivation gain is expected for the patterned samples, as both have an equal nominal passivation area coverage. The higher effective gain in sample F8, in the same order as the JV gain, further showcasing the potential of this near‐total rear passivation scheme. Moreover, the V_OC_ enhancement is in the upper range with respect to the ones reported in the literature, indicating an effective passivation of recombination mechanisms, especially considering that all devices have approximately the same bandgap energy value around 1.32 eV, obtained through the first derivative of the EQE curves, with the values shown in Table 1 [26, 65]. To gain deeper insight into the recombination mechanisms and diode behavior of the fabricated devices, a one‐diode model fit was applied to the light JV curves, following Hegedus et al. [66], enabling extraction of advanced electrical parameters including the ideality factor (A), saturation current density (J_0_), shunt resistance (R_shunt_), and series resistance (R_S_) that allow a more comprehensive assessment of junction quality and recombination pathways across the different passivation strategies. The obtained A value is similar across all devices, with a value around 1.5 – 1.7, showing the consistency of the developed ACIGS absorbers. The obtained values suggest that dominant recombination mechanisms can be described by the Shockley–Read–Hall (SRH) model [67]. With respect to J_0_, the higher value is obtained for the REF sample, suggesting recombination is in fact reduced with the implementation of any of the studied passivation strategies. Regarding the R_shunt_ parameter, an increase is obtained for all passivated samples, regardless of the architecture, with respect to the REF one. Notably, the higher shunt resistance (R_shunt_) average value is attained for the F8 sample, in which the larger passivated area was designed. A reduction of shunting paths, seen through a larger R_shunt_, has already been previously reported for ultra‐thin devices emplyoing a dielectric passivation strategy, and this has been attributed to a reduction of the rear interface conductive area [19, 22, 24, 68, 69]. Such an increase in R_shunt_ is important as with the thinning down of the absorber, the probability of increased shunts increases, and therefore the dielectric layer brings additional benefits other than just of passivation. Finally, for R_S_, this parameter may increase in the passivated samples since we are adding a “non‐conducting layer” to the rear interface; however, a nontrivial trend is observed. Although no significant variations are obtained in the R_s_ values between all samples, comparing the REF and the patterned passivated samples, P8 and P25, the R_S_ does increase but only slightly; for the sample F8, the R_S_ decreases, being the lowest. Such a decrease in the R_S_ suggests that no barrier blocking the charge carriers exists and that the porosity may have enabled pathways for efficient charge extraction. Therefore, this in‐depth one‐diode JV analysis supports that the observed electrical improvements stem from the passivation effects at the rear interface. However, in the case of the full SiO_x_ layer, which achieved the best performance, the origin of the lower R_S_ is still unclear. Regarding FF, since no significant differences are attained in the R_S_ value, it follows the R_shunt_ one, with the highest FF average value of 76.2% being obtained for the F8 sample, and the lowest of 74% for the REF one. Looking into J_sc_ values, a maximum increase of around 0.6 mA**·**cm^−2^ is obtained for the P25 sample with respect to the REF one. The representative EQE data in Figure 5B and the ACIGS absorptance spectra presented in Figure 5C, obtained from FDTD 3D optical simulations, were used to decouple the passivation electronic effect from the optical improvement. In both measured and simulated EQE, a slightly higher EQE in the passivated samples from the REF one arises in the spectral region from 600 nm to the Near‐Infrared. Such an increase occurs due to an improved rear reflection from the difference between CIGS‐based (3.01 at 600 nm) and SiO_x_ (1.47 at 600 nm) n values [20, 26, 70]. Overall, the simulated optical gain is small, only visible on the two peaks in the 750 – 900 nm region, and the introduction of any full or pattern SiO_x_ layer should give a slight maximum gain in J_sc_ of 0.2 mA**·**cm^−2^. Hence, the overall J_SC_ enhancement obtained in the JV measurements is attributed to improved rear interface defect passivation. Finally, a statistical representation of the figures of merit obtained from the JV characteristic curves is presented in Figure 5D, highlighting the enhancements obtained with the passivation strategies, more predominant with the full‐layer coverage (F8). Moreover, the figures of merit of each sample have a small spread around the average value, as also seen by the standard deviation values in Table 1.

**TABLE 1:** Average (with standard deviation) and best values of the figures of merit from 16 solar cells per device, based on JV data. The advanced parameters R_shunt_, R_s_, A, and J_0_ were derived using a one‐diode model fit.

		V_OC_ (mV)	J_SC_ (mA·cm^−2^)	FF (%)	η (%)	J_0_ (nA·cm^−2^)	A	R_shunt_ (kΩ·cm^2^)	R_s_ (Ω·cm^2^)	E_g_ (eV)
**REF**	Mean	690 ± 7	27.6 ± 0.3	74.0 ± 0.5	14.1 ± 0.3	3.18 ± 2.92	1.70 ± 0.1	0.97 ± 0.12	0.70 ± 0.12	1.31
	Best	699	28.1	74.4	14.6					
**F8**	Mean	749 ± 10	28.1 ± 0.3	76.2 ± 0.4	16.0 ± 0.3	1.90 ± 1.55	1.72 ± 0.09	2.24 ± 0.14	0.66 ± 0.06	1.32
	Best	759	28.5	76.0	16.4					
**P8**	Mean	734 ± 4	28.1 ± 0.2	75.8 ± 0.7	15.6 ± 0.2	0.96 ±0.49	1.49 ± 0.35	1.91 ± 0.10	0.86 ± 0.06	1.32
	Best	736	28.4	76.7	16.0					
**P25**	Mean	721 ± 11	28.2 ± 0.3	75.1 ± 0.7	15.3 ± 0.3	1.36 ± 0.82	1.66 ± 0.06	1.55 ± 0.40	0.85 ± 0.09	1.32
	Best	737	28.2	76.0	15.8					

**FIGURE 6:**
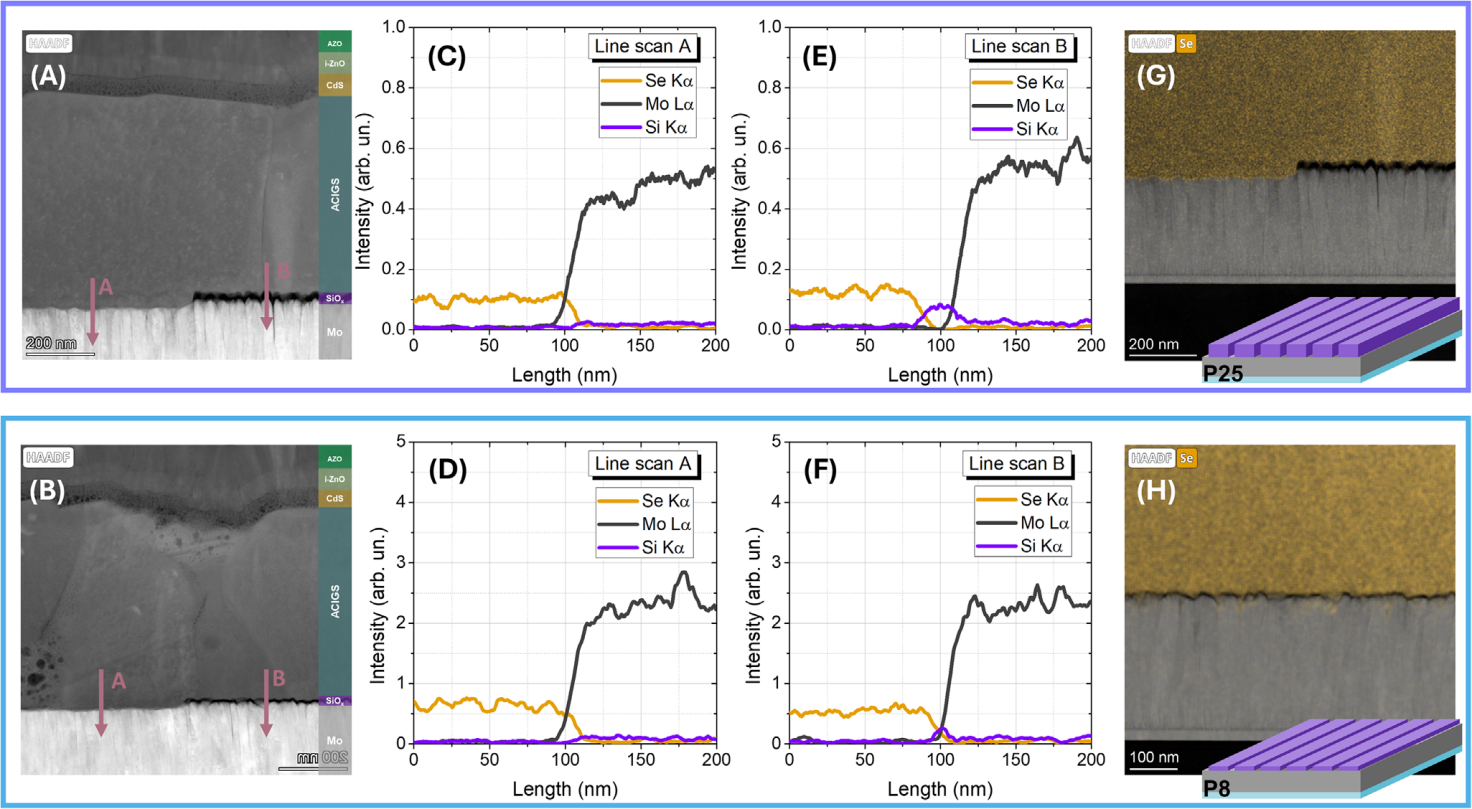
STEM HAADF images of (A) P25 and (B) P8 solar cells. Each image presents two studied line profiles (pink arrow): A and A for both P8 and P25, corresponding to the Mo/ACIGS and Mo/SiO_x_/ACIGS interfaces, respectively. EDS line profiles for Se, Mo, and Si elements of line scan A of samples (C) P25 and (D) P8, and of line scan B of sample (E) P25 and (F) P8. EDS maps of Se distribution for (G) P25 and (H) P8.

Despite the measured Si signal in the GDOES measurements, there is still no definitive proof that the SiO_x_ passivation schemes are fully intact following the ACIGS deposition. Moreover, a more in‐depth analysis of the contact nature is also key to better understand the solar cells' performance. Thus, to assess the SiO_x_ layer integrity and the contact characteristics, a lamella of each of the passivated devices was prepared through FIB, and a STEM analysis was performed. While for the STEM only one lamella was studied, the complete near 5 µm side length was scrutinized to ensure the observations were uniform. In the STEM images HAADF of Figure 6A,B is possible to see that for the line pattern passivated samples, P25 and P8, the SiO_x_ lines are intact, with a thicker layer on the P25 sample. Moreover, the CdS buffer layer and both ZnO_x_‐based layers of the optical window are also visible, with a uniform coverage over the ACIGS absorber. In a standard Mo/CIGS interface, the selenization of the Mo surface that occurs during the first stages of the ACIGS deposition leads to the MoSe_2_ layer formation [26]. In Figure 6A,B, two line scans in the contact line interface region were performed: line scan A in P8 and P25, being the EDS line profiles for Se, Mo, and Si elements shown in Figure 6C,D, respectively. There is a clear overlap of the Mo and Se signals, suggesting the formation of the MoSe_2_ layer within the line contacts of the passivated samples. It is noteworthy that it has been demonstrated that this layer facilitates the formation of a quasi‐ohmic type contact between the CIGS and Mo [71, 72]. Furthermore, it is expected to find this layer only at the contact openings, and not where the SiO_x_ was deposited. The EDS line scans performed in the passivation region, shown in Figure 6E,F, line scan B in P25 in Figure 6A and in P8 in Figure 6B, present a peak associated with the passivation line and no clear Se and Mo signal overlapping, so no evidence of MoSe_2_. A Se elemental distribution EDS mapping was also conducted in both the P8 and P25 samples. In Figure 6G, the Se mapping of sample P25 corroborates the line scan B profile of sample P25. However, in sample P8, Figure 6H, several spots emerge with Se below the SiO_x_, indicating Se penetration through the porous passivation layer.

**FIGURE 7:**
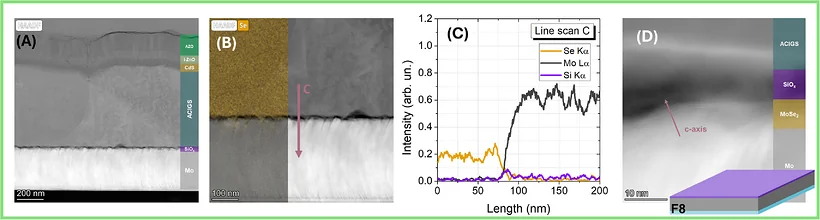
STEM HAADF images of (A) F8 solar cell, and (B) the rear interface of F8 solar cell with an EDS map of Se distribution and one line profile (pink arrow) C corresponding to the Mo/SiO_x_/ACIGS interface. (C) EDS line profiles for Se, Mo, and Si elements for line scan C. On (D), a high‐magnification STEM image shows the Mo/MoSe_2_(SiO_x_)/ACIGS interface.

Concerning the sample with a full 8 nm passivation layer, F8, the lower magnification STEM HAADF image in Figure 7A shows the existence of a uniform SiO_x_ layer across the sample. Although by close inspection at higher magnification Figure 7B, small, blurred spots appear within the SiO_x_ layer, which may be associated with a higher‐porosity SiO_x_ region where there is a higher MoSe_2_ formation probability. Similar to the P8 sample (Figure 6H), in these blurred spots, the Se elemental distribution bypasses the SiO_x_ layer and reaches the Mo, where it may form MoSe_2_. The line scan in Figure 7C, performed at the rear interface of sample F8 in the region of Figure 7B, reveals the Mo, Se, and Si distribution in a spot where the Se distribution does not cross the SiO_x_ layer to the Mo; hence, supposedly a lower porosity region, and so no clear Se and Mo overlapping is observed. In the high‐magnification STEM image of the interface below the SiO_x_ layer, where pores are probably present – Figure 7D, the characteristic layered structure of MoSe_2_ with a *c*‐axis orientation perpendicular to the Mo interface was detected [73, 74, 75]. Despite being recognizable at close, the crystal structure has low contrast. This can have happened due to surface amorphization during the FIB lamella fabrication, but also because these are local size formations that, due to the small thickness of the passivation layer and the roughness of the Mo rear contact, do not have a lateral and depth wise uniform distribution.

It is suggested that the porosity of the full passivation layer is the key characteristic that enables the highest‐performance device, by decreasing recombination at the rear interface and still allowing charge transport to occur in a type‐ohmic contact formed with MoSe_2_. Alkali doping, in this case by diffusion of Na from the SLG and the pre‐deposition treatment (DT) of NaF, is a key element for high‐performance CIGS‐based solar cells [62, 76], and it has been demonstrated that Na is a driving force in the formation of MoSe_2_; when no Na source is present, the MoSe_2_ formation is seriously hindered [51, 77]. Ledinek et al. showed the importance of Na when having a full passivation layer of AlO_x_: when no source of Na exists, the solar cells exhibit a blocking behavior; also, if only a post‐DT is applied (after de CIGS growth), the performance is still hindered if a thick passivation layer is used. However, when Na is supplied and free to diffuse, the solar cell can benefit from enhanced performance via rear passivation [51]. These have been tested in Al_2_O_3_ and HfO_2_, and in both, a critical possibility of Na damaging the passivation layer arises; although no clear holes in the layer are observed [51, 78]. Nevertheless, Na supply is necessary to enable conductivity through the full passivation layers, although being a compromise between the benefits of Na and the rear passivation area coverage [78]. In previous works, when the alkali metals are supplied by a spin‐coated alkali fluoride on top of the passivation layer, the strategy developed is to intentionally perforate the passivation layers with alkali diffusion; the openings are randomly distributed and allow direct contact between the CIGS and the Mo while maintaining a passivation area above 95% [79, 80, 81]. Lastly, it can be stated that Na doping is essential for the high performance of the solar cell with the full passivation layer, since it may facilitate the formation of MoSe_2_ through the porous passivation layer.

**FIGURE 8:**
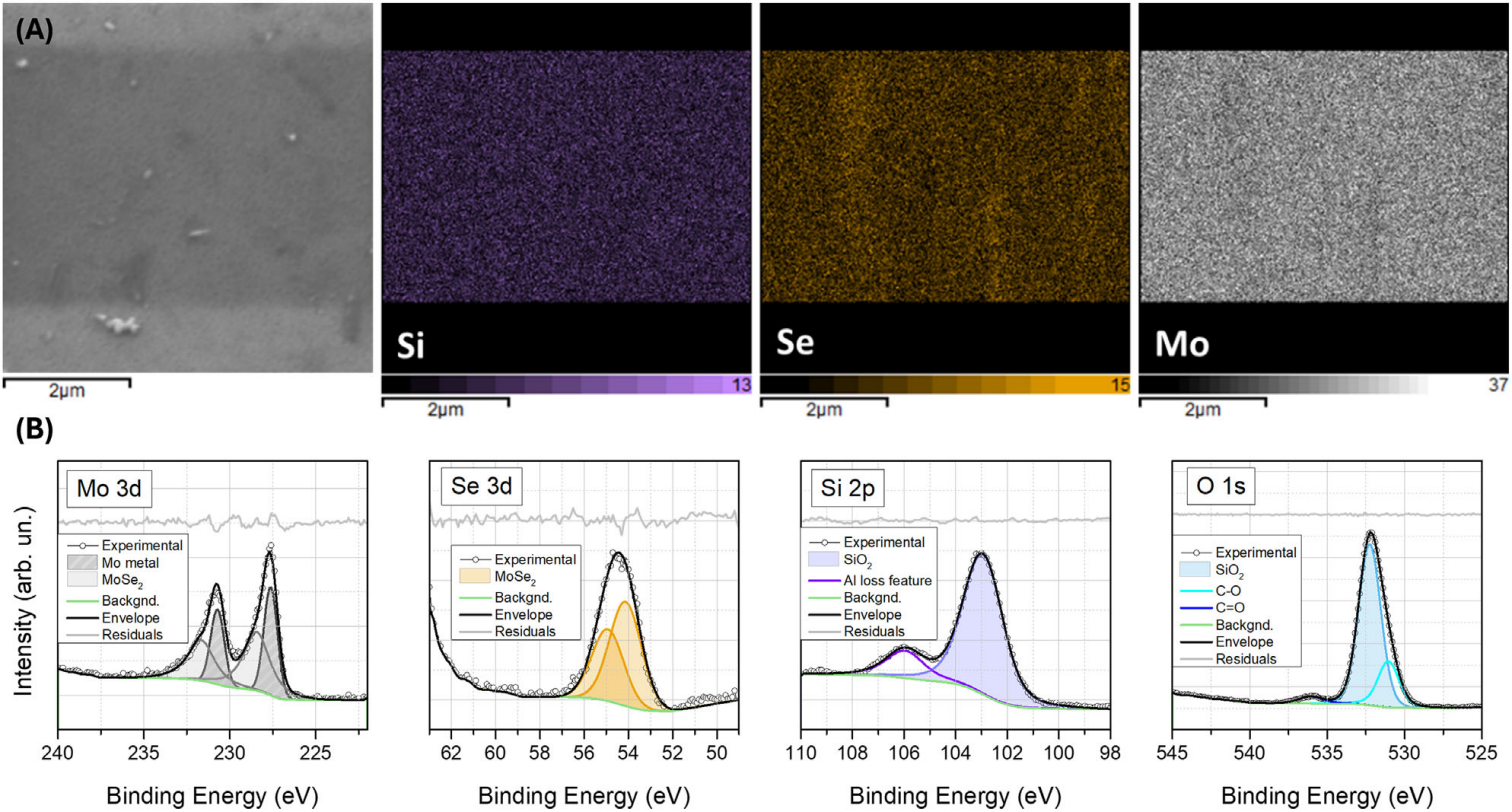
After peel off (A) SEM EDS maps for the F8 sample. The Si distribution was obtained with a Kα 1 emission line, Se with Lα 1 and 2, and Mo with Lα 1, and (B) surface XPS elemental analysis spectra of Mo 3d, Se 3d, Si 2p, and O 1s.

STEM‐EDS shows that all rear passivation strategies are locally intact, and the 8 nm SiO_x_ layer in samples P8 and F8 allows the diffusion of Se and so the formation of MoSe_2_ below the passivation layer. To further investigate if this result extends across all area of sample F8, a peel‐off procedure was done to perform a top‐view analysis of a larger area of the rear interface. Such a procedure was performed as reported elsewhere [82], with an extra cooling step with liquid nitrogen between the epoxy curing and the cleavage step. After such a procedure, two distinct sides of the sample were inspected: (i) the metallic sheet with the ZnO:Al/i‐ZnO/CdS/ACIGS structure (from bottom to top) and (ii) the original SLG with the Mo rear contact, the MoSe_2,_ and the passivation layer. In Figure 8A, a top‐view SEM image of the rear surface and corresponding Si, Se, and Mo elemental distribution EDS maps are presented for side (ii). The SiO_x_ layer is intact, even after a peel‐off procedure. Across the surface, there are several debris that is believed to be remnants of the peel‐off procedure, including the darker spots. Nevertheless, the SiO_x_‐coated Mo rear contact has an as‐deposited appearance, showing that it was not damaged in the harsh ACIGS deposition. The Si elemental distribution across the rear interface surface (Figure 8A) displays a uniformly distributed SiO_x_ layer with no interruption; also, the Se distribution is fairly uniform besides often accumulating in some areas. The Se distribution and its accumulation cannot be correlated with any change in the Si signal or any significant morphological modification of the rear interface. This SEM mapping analysis clearly shows the uniform, non‐localized distribution of MoSe_2_ across the rear interface of sample F8. Nevertheless, to further confirm its existence and to investigate what is the chemical state of these elements, XPS surface analysis was performed. The atomic percentage values for each of the acquired elements are listed in Table 2, and the elemental orbital spectra of Mo 3d, Se 3d, Si 2p, and O 1s are presented in Figure 8B. Note that contamination due to handling, such as carbon, was not considered in this calculation. The quantification reveals that 18.4% originates from ACIGS remnants due to peel‐off at the rear interface, with the majority of this contribution attributed to Ga, likely a result of the higher Ga profile implemented at the rear interface, and the Na signal is attributed to unreacted NaF from the pre‐deposition treatment. Considering first the Si 2p and O 1s spectra associated with the SiO_x_ layer, the Si 2p spectrum exhibits two distinguishable peaks: one attributed to SiO_2_ at 103 eV, and the other to an aluminium loss feature, likely an artifact from the x‐ray source gun. The O 1s spectrum, in turn, shows three peaks, with the most intense attributed to SiO_2_ at 532.3 eV, while the remaining two are assigned to single and double carbon bonds (O─C and O═C, respectively). Considering the atomic percentages of the two peaks associated with SiO_2_ (Si 2p and O 1s), a stoichiometric ratio of 2 was calculated, consistent with and confirming the presence of the passivation layer. Second, the Mo 3d spectrum reveals two doublets: one associated with Mo in its metallic state (227.6 eV) corresponding to the rear contact, and the other associated with a Mo–Se bond (228.4 eV). In the Se 3d spectrum, only one doublet is observed, also associated with a Mo–Se bond (54.1 eV). Considering the atomic percentages of the peaks associated with the Mo–Se bond, a stoichiometric ratio of 2.1 was calculated, confirming the presence of the MoSe_2_ layer. This latter value should be regarded as an estimation, since the average XPS probing depth is approximately 10 nm, while the passivation layer itself is 8 nm thick—meaning only the outermost 2 nm below the SiO_x_ layer were effectively probed. Overall, the XPS analysis suggests an intact SiO_2_ passivation layer and a rather uniform MoSe_2_ layer below it. Thus, the high‐performance enhancement can be related to the combination of near‐full passivation, translated into the highest R_shunt_ and a near 60 mV V_OC_ increase, and the formation of MoSe_2_ through the pores, leading to an ohmic contact, resulting in the lowest R_s_, leading to an average increase of almost 2% absolute efficiency.

**TABLE 2:** Atomic percentages obtained through x‐ray photoelectron spectroscopy surface analysis at the rear interface of sample F8 after peel‐off, and corresponding chemical state.

Si	O	Mo		Se	Ag	Cu	In	Ga	Na
SiO_2_	SiO_2_	metal	Mo–Se	Mo–Se	—	—	—	—	—
25.0%	50.9%	1.1%	1.4%	3.0%	0.3%	0.5%	9.2%	1.2%	7.3%

## 4 Conclusions

Interface passivation has been a key driving force for performance improvements in solar cells, both for Si and CIGS‐based technologies, with patterned dielectric layers and tunnel oxide layers as key strategies. In the present study, the incorporation of a porous rear passivation layer with full coverage in ultra‐thin ACIGS solar cells is tested. Three passivated samples were developed: two line‐patterned, with around 70% of the passivated area but different SiO_x_ thicknesses, 8 and 25 nm (P8 and P25), and one porous full, i.e., 100% passivation area, layer of 8 nm (F8). The samples were fully characterized by GDOES depth profiling, JV, EQE, and electron microscopy (SEM and STEM). First, GDOES and STEM analysis proved that all passivation architectures survived the harsh ACIGS deposition conditions. Then, performance‐wise, the full‐layer passivation sample (F8) has the highest efficiency, with a 2% absolute value increase and a maximum value of 16.4%. Also, both the V_OC_ and FF were enhanced, which has been associated with a passivation effect since the optical gain is negligible, as seen through EQE and supported by optical simulations. The remaining passivated samples also had performance enhancements, but to a smaller extent. After One‐diode fitting, sample F8 was found to have the highest shunt resistance and the smallest series resistance. In the STEM analysis, both patterned samples have the characteristic MoSe_2_ of the Mo/CIGS interface in the contact openings. For the samples with an 8 nm passivation layer, patterned (P8) and full (F8), the MoSe_2_ was found below the passivation layer. A peel‐off procedure of sample F8 allowed a deeper assessment of the rear interface and showed that the full SiO_x_ layer was found uniform, intact, and with no morphological deformation. Moreover, XPS analysis of this rear contact confirmed the existence of SiO_2_ and MoSe_2_, both with a stoichiometric ratio of 2. Summarizing, the high performance of the full porous passivated sample was attributed to the near‐complete rear interface passivation, since no defects were found in this layer, combined with the type‐ohmic of Mo/MoSe_2_(SiO_x_)/ACIGS across the rear interface. The diffusion of Na and Se through pores of the passivation layer is associated as the most probable cause of the MoSe_2_ formation.

## Author Contributions

**Xavier L. Pinheiro**: writing – original draft, investigation, visualization, formal analysis, data curation, methodology. **António J. N. Oliveira**: writing – review and editing, writing – original draft, software, methodology. **André F. Violas**: writing – review and editing, validation, methodology. **Kevin Oliveira**: writing – review and editing, investigation, validation. **Paulo A. Fernandes**: writing – review and editing, funding acquisition, resources. **Marika Edoff**: writing – review and editing, project administration, resources. **Pedro M. P. Salomé**: writing – review and editing, conceptualization, funding acquisition, supervision, project administration, resources. **Jennifer P. Teixeira**: conceptualization, investigation, funding acquisition, writing – review and editing, supervision, methodology, project administration.

## Funding

This work was supported by the Innovation Pact “R2UTechnologies—modular systems,” with the reference number C644876810‐00000019. The work of Xavier L. Pinheiro and Jennifer P. Teixeira was supported by Fundaçao para a Ciencia e Tecnologia (FCT) under grants number 2022.14053.BD and 2021.02405.CEECIND, respectively.

## Conflicts of Interest

The authors declare no conflicts of interest.

## Data Availability

The data that support the findings of this study are available from the corresponding author upon reasonable request.
